# Women’s contribution to medicine in Bahrain: leadership and workforce

**DOI:** 10.1186/s12960-022-00762-9

**Published:** 2022-09-05

**Authors:** Feras H. Abuzeyad, Leena Al Qasem, Luma Bashmi, Mona Arekat, Ghada Al Qassim, Ahmed Alansari, Eman Ahmed Haji, Amena Malik, Priya Das, Abdulla Almusalam, Maryam Feras Abuzeyad

**Affiliations:** 1 Emergency Medicine, King Hamad University Hospital, Building 2435, Road 2835, Block 228, P.O. Box 24343, Busaiteen, Kingdom of Bahrain; 2 Riffa, Kingdom of Bahrain; 3 Department of Health Psychology, School of Medicine, Royal College of Surgeons in Ireland - Bahrain, Busaiteen, Kingdom of Bahrain; 4 Internal Medicine Department, Arabian Gulf University, Manama, Kingdom of Bahrain; 5 Emergency Medicine Department, Bahrain Defence Force Hospital, Royal Medical Services, Riffa, Kingdom of Bahrain; 6 Department of General Surgery, Bahrain Defence Force Hospital, Royal Medical Services, Riffa, Kingdom of Bahrain; 7 Ministry of Health and Social Welfare, Manama, Kingdom of Bahrain; 8 College of Business and Aviation, Fairmont State University, Fairmont, USA; 9 Research Department, King Hamad University Hospital, Busaiteen, Kingdom of Bahrain

**Keywords:** Women, Medicine, Leadership, Health workforce, Medical students, Gender

## Abstract

**Background:**

Women make up a significant proportion of workforce in healthcare. However, they remain underrepresented in leadership positions relating to healthcare for a multitude of reasons: balancing personal and work duties, favoritism toward men, lack of support from colleagues and mentors, as well as other factors. This study aims to recognize the contribution made by women in the Bahraini healthcare sector by determining the gender distribution in Bahrain’s medical schools, government hospitals, Ministry of Health, and National Health Regulatory Authority.

**Methods:**

Data were collected from the Bahraini Ministry of Health, National Health Regulatory Authority, Salmaniya Medical Complex, King Hamad University Hospital, Bahrain Defence Force Royal Medical Services, the College of Medicine and Medical Sciences in the Arabian Gulf University, and the Royal College of Surgeons in Ireland-Bahrain. Only physicians who held a Bachelor of Medicine and Surgery and a valid license to practice from NHRA were eligible to participate. Descriptive statistics were used to derive the frequencies and percentages of physicians with the following leadership positions: (1) top administrative positions (e.g., Chief executive officer); (2) heads of departments; (3) heads of committees; and (4) academic positions (e.g., Professor). Data were also collected from the two medical schools in Bahrain to see the trend in female enrollment into medical schools since 2004.

**Results:**

The results of the study indicated that leadership positions were mostly held by males in Bahrain (59.4% vs. 40.6%). However, Bahraini males and females equally dominated academic positions. Male physicians also dominated surgical specialties; however, female Bahraini physicians slightly surpassed male Bahraini physicians at the specialist and consultant levels (female to male: 11.9% vs. 10.4% and 33.2% vs. 30.4%, respectively). Furthermore, more females were reported to have general licenses. A trend analysis since 2004 showed that female medical students’ representation was higher than males over the years.

**Conclusions:**

This study highlights the increasing trend of women’s participation and contribution to medicine in Bahrain. The data indicated continued growth in the number of female medical students and physicians. As such, it is likely that females will have a bigger impact on healthcare in the future with potential to hold more leadership positions in Bahrain.

**Supplementary Information:**

The online version contains supplementary material available at 10.1186/s12960-022-00762-9.

## Introduction

Throughout history, women have fought and made great sacrifices to study and practice medicine to achieve their current status [1, 2]. The World Health Organization (WHO) reported data from 104 countries revealing that women make up to 70% of the workforce in health and social areas [3]. In the last 15 years, the Organization for Economic Co-operation and Development (OECD) announced a significant rise in the number of female physicians working in the health sector [4]. The trend of women dominating the medical workforce has led to the term “feminization of medicine” [5]. Despite this major gender turnover in the medical college pipeline, women are still underrepresented in leadership positions, especially in academic medicine [6]. The slow progress of women in leadership positions in the medical field has led to the well-known concept, the “glass ceiling” effect, which represents any barriers preventing class workers, especially women, from reaching top positions [7]. This concept could be due to multiple key elements related to women being perceived as not being fully committed to the workplace, including: (1) balancing family vs. work duties including raising children, home activities, and working part-time; (2) social and cultural expectations from women; (3) favoritism toward men over women in the market by their organizations; and (4) and lack of support in the form of mentors, role models, work flexibility, and other factors [7, 8]. A balanced distribution of a country’s healthcare workforce is an essential demand by the WHO to accomplish several Sustainable Development Goals, which include gender equality [9]. Several studies have shown that women physicians provide more patient-focused communication, more evidence-based management, better care for diabetic patients, lower mortality rates, readmission to hospitals, and emergency department visits [8]. Women have contributed to the advancement of medicine even before the nineteenth century [10], and this is particularly true for the Kingdom of Bahrain.

This study aimed to recognize the contribution of women to medicine in the leadership and workforce in the Kingdom of Bahrain, taking into consideration that no previous research was done as far as we know in the context of the medical field. The study objectives were to determine the estimated gender distribution figures in Bahrain’s medical schools, government hospitals, healthcare centers, and National Health Regulatory Authority (NHRA) for the following variables in 2020: (1) physician workforce; (2) physician leadership; (3) physician workforce according to medical specialty; and (4) medical education workforce and medical students since 2004.

### History of women in medicine in Bahrain

Bahrain was the first country in the Arabian Gulf to set a conducive environment for women’s contribution to the medical workforce. In the early twentieth century, Dr. Marion Wells Thomas was the first female physician to practice modern medicine in Bahrain after she arrived with her husband Dr. Sharon J Thomas in 1900. Dr. Thomas worked for the American missionary at Mason Memorial Hospital, which was opened in 1903 as the first missionary hospital in Arabia [11]. Later in 1938, Dr. M. Mc Dowell arrived in Bahrain to work as a senior obstetrician, where she left the country in 1942 and was replaced by another female physician, Dr. I. Doeg. In 1947, Dr. Mary Abraham arrived from India and joined the maternity section [12]. In parallel to this, Bahrain was also the first country in the Arabian Gulf to open a public school for girls in 1928, and in the early 1950s, Bahraini women benefited from governmental scholarships for higher education to study medicine in Egypt, Lebanon, Iraq, and Syria [13]. The first Bahraini female to acquire a bachelor’s degree in medicine was Dr. Sadeeqa Ali Al-Awadi in 1969 [14]. Since then, the number of Bahraini female physicians increased exponentially in the country’s workforce, with women taking on multiple leadership roles. This was followed by the opening of the first medical school in 1984 in Bahrain, the “College of Medicine and Medical Sciences”, which allowed for even more opportunities for females to contribute to and excel in medicine [15]. Bahraini Dr. Naeema Hassan Al-Qaseer, was the first female primary care physician to be appointed as an expert in the WHO in 1996 [14]. Bahrain has always viewed women as equal partners to men in the workforce. With the establishment of the Supreme Council for Women in 2001 upon Amiri order No. 44, the country’s emphasis on enabling more women to hold leadership positions increased [16]. In 2002, female Bahraini medical students comprised 63% of the total enrolled number at AGU, and women contributed a total of 55% to the healthcare workforce [13]. The second medical college, the Royal College of Surgeons in Ireland (RCSI), opened its medical college in Bahrain, the “Medical University of Bahrain”, or RCSI–MUB in 2004 [17]. Dr. Nada Haffadh was the first female physician to be appointed as the Minister of Health in 2004 [13, 14]. In 2012, Dr. Maha Al-Kuwari was elected as the first female president of Bahrain Medical Society [18].

More recently in 2020, Bahrain’s Supreme Council for Women initiated new policies during the COVID-19 pandemic to mitigate its impact on women. Three policy changes have occurred to support the ‘gender balance’ approach including: (1) prioritizing remote work for female frontline healthcare workers and setting aside the first hour of service operation for pregnant women and the elderly; (2) encouraging and providing psychological counselling virtually; and (3) extending loan and debt waiver to women with outstanding debts. These newly appointed policies further highlight the priority the Kingdom of Bahrain gives to the female workforce in the context of the COVID-19 pandemic [19].

## Methods

This is a multi-center retrospective case study in which a pre-set data sheet was constructed, and information was gathered from the participated institutions looking at the current leadership, faculty, and medical student and workforce figures for the year 2020 in the Kingdom of Bahrain. The following institutions took part in the study: Ministry of Health (MOH), National Health Regulatory Authority (NHRA), Salmaniya Medical Complex (SMC), King Hamad University Hospital (KHUH), Bahrain Defence Force Royal Medical Services (BDF–RMS), the College of Medicine and Medical Sciences in the Arabian Gulf University (AGU), and the Royal College of Surgeons in Ireland–Bahrain (RCSI–MUB). Ethical approvals were obtained from the ethical boards of all participating institutions.

Data were collected from the participants’ administrative databases for the year 2020. To be considered, practicing physicians must hold a Bachelor of Medicine and Surgery and a valid license from NHRA. Physicians who hold leadership position(s) were either practicing physicians who hold administrative positions in addition to their practice, or non-practicing physicians holding administrative positions. Nurses, pharmacists, dentists, technicians, and other allied health care providers were not included. Medical students enrolled in the study were from the two existing medical schools in Bahrain; AGU and RCSI–MUB.

Data that fit the inclusion criteria was split according to gender (female/male) and citizenship (Bahraini/non-Bahraini) and pooled into three main categories:

A. Leadership positions,
B. Licensed physicians by NHRA, and
C. Graduated medical students from the two medical schools.

The following were considered as leadership positions:

1. Top administrative positions (e.g., minister, chief executive officer, chief of staff, training director, etc.),
2. Heads of departments,
3. Heads of committees, and
4. Academic positions (e.g., professor, associate professor, assistance professor, etc.).

Within the physician workforce, data was further split according to licensing category (consultants, specialists, or general physicians), medical specialty, and surgical specialty. NHRA, which is the licensing authority in Bahrain, licenses physicians into three levels: general license (physicians who are at the level of a resident or a senior resident and have not obtained a recognized specialization certificate yet), specialist licenses (physicians who hold recognized specialization certificates with a certain number of years of experience post qualification) and consultant licenses (physicians with recognized specialization certificates with a certain number of years of experience post qualification). Finally, the graduated medical students were split according to year of graduation.

### Statistical analysis

Data was analyzed using the Statistical Package for Social Sciences software (SPSS, V.25; International Business Machines, Chicago, IL). Descriptive statistics were used to derive the frequencies and percentages. A Chi square test was used to compute any significant differences among genders. Trend analysis was done to analyze the change in number of medical students over the years. Statistical significance was defined as a *p* value of less than 0.05.

## Results

### Leadership

**Fig. 1:**
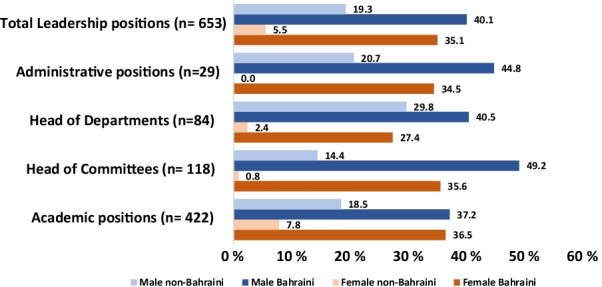
Leadership positions by gender and citizenship

Of the 653 leadership positions in Bahrain, 388 (59.4%) were held by males and 265 (40.6%) were held by the females. Bahraini females dominated the majority of leadership positions compared to non-Bahraini females (*p* = 0.023). The leadership positions were almost equally dominated by female and male Bahrainis (*p* = 0.40) (Table 1 and Fig. 1).

**Table 1:** Leadership positions breakdown by gender and citizenship

Leadership Positions	Female (%)		Male number (%)		Total
	Bahraini	Non-Bahraini	Bahraini	Non-Bahraini	
Administrative Positions	10 (34.4%)		19 (65.6%)		29
	10 (34.5%)	0 (0%)	13 (44.8%)	6 (20.7%)	
Head of Departments	25 (29.7%)		59 (70.3%)		84
	23 (27.4%)	2 (2.4%)	34 (40.5%)	25 (29.8%)	
Head of Committees	43 (36.4%)		75 (63.6%)		118
	42 (35.6%)	1 (0.8%)	58 (49.2%)	17 (14.4%)	
Academic Positions	187 (44.3%)		235 (55.7%)		422
	154 (36.5%)	33 (7.8%)	157 (37.2%)	78 (18.5%)	
Total	265 (40.6%)		388 (59.4%)		653
	229 (35.1%)	36 (5.5%)	262 (40.1%)	126 (19.3%)

### Physician workforce

**Fig. 2:**
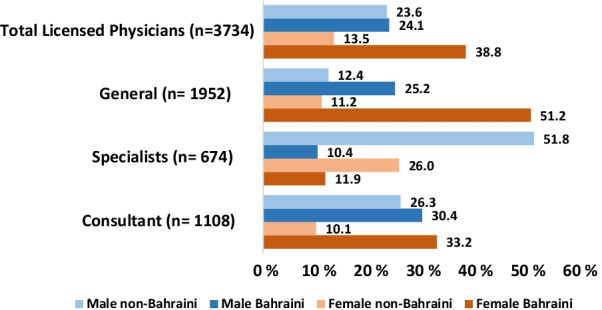
Licensing categories by gender and citizenship

NHRA reported 3734 licensed physicians in Bahrain, which included 1108 (29.6%) consultants, 674 (18.05%) specialists and 1952 (52.2%) physicians with general licenses. The total number of male physicians constituted 1782 (47.8%), while females were 1952 (52.2%) of the workforce in Bahrain (Table 2 and Fig. 2).

**Table 2:** Licensing categories breakdown by gender and citizenship

Licensing categories	Female number (%)		Male number (%)		Total
	Bahraini	Non-Bahraini	Bahraini	Non-Bahraini	
Consultant	480 (43.3%)		628 (56.7%)		1108
	368 (33.2%)	112 (10.1%)	337 (30.4%)	291 (26.3%)	
Specialist	255 (37.8%)		419 (62.2%)		674
	80 (11.9%)	175 (26.0%)	70 (10.4%)	349 (51.8%)	
General	1217 (62.3%)		735 (37.7%)		1952
	999 (51.2%)	218 (11.2%)	492 (25.2%)	243 (12.4%)	
TOTAL	1952 (52.2%)		1782 (47.8%)		3734
	1447 (38.7%)	505 (13.5%)	899 (24%)	883 (23.8%)

**Fig. 3:**
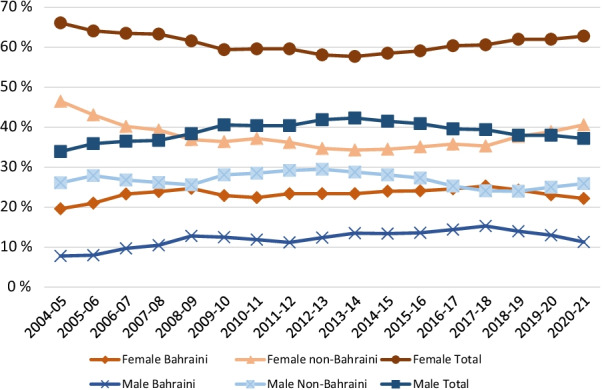
Percentage of medical students graduates in Bahrain by gender and citizenship

There was a higher number of females with general licenses compared to males (62.3% vs. 37.7%), especially when compared to the gender distribution of consultants (43.3% vs. 56.7%, *p* = 0.02) and specialists (37.8% vs. 62.2%, *p* = 0.00), both of which were dominated by males (Table 2 and Fig. 2). Although most consultants and specialists were male, specialties such as family medicine (75.7% vs. 24.3%, *p* = 0.00) and Obstetrics and Gynaecology (87.9% vs. 12.1%, *p* = 0.00) were dominated by females (Table 3). Within medical and surgical specialties (represented by consultants and specialists only), males dominated all medical (except for clinical geneticist, 100% female, n = 3; Table 3 and Additional file 1: Annex 1) and surgical specialties (*p* = 0.00) (Additional file 2: Annex 2).

**Table 3:** Specialty categories (consultants and specialists) breakdown by gender and citizenship

Specialty	Female number (%)		Male number (%)		Total = 1782
	Bahraini	Non-Bahraini	Bahraini	Non-Bahraini	
Internal Medicine (including all subspecialties)	124 (34.1%)		240 (65.9%)		364
	50 (13.7%)	74 (20.4%)	89 (24.4%)	151 (41.5%)	
Family medicine	199 (75.7%)		64 (24.3%)		263
	194 (73.7%)	5 (2%)	55 (20.9%)	9 (3.4%)	
Surgery (including all surgical subspecialties)	67 (19.1%)		285 (80.9%)		352
	46 (13%)	21 (6.1%)	122 (34.6%)	163 (46.3%)	
Orthopaedics	5 (5.9%)		80 (94.1%)		85
	2 (2.3%)	3 (3.6%)	27 (31.7%)	53 (62.4%)	
Obstetrics and gynaecology	123 (87.9%)		17 (12.1%)		140
	50 (35.7%)	73 (52.2%)	4 (2.8%)	13 (9.3%)	
Anaesthesia	35 (26.8%)		96 (73.2%)		131
	6 (4.5%)	29 (22.3%)	12 (9.1%)	84 (64.1%)	
Radiology	39 (41.1%)		56 (58.9%)		95
	13 (13.6%)	26 (27.5%)	10 (10.5%)	46 (48.4%)	
Emergency medicine	8 (24.3%)		25 (75.7%)		33
	7 (21.2%)	1 (3.1%)	13 (39.3%)	12 (36.4%)	
Psychiatry	21 (43.8%)		27 (56.2%)		48
	15 (31.2%)	6 (12.6%)	20 (41.6%)	7 (14.6%)	
Paediatrics	76 (43.2%)		100 (56.8%)		176
	47 (26.7%)	29 (16.5%)	43 (24.4%)	57 (32.4%)	
ICU	0 (0%)		14 (100%)		14
	0 (0%)	0 (0%)	1 (7.1%)	13 (92.9%)	
Oncology	8 (27.6%)		21 (72.4%)		29
	5 (17.2%)	3 (10.4%)	6 (20.6%)	15 (51.8%)	
Laboratory medicine	30 (57.7%)		22 (42.3%)		52
	13 (25%)	17 (32.7%)	5 (9.6%)	17 (32.7%)	

## Medical students

Trend analysis indicated that there were more female medical students than males over the years (Additional file 3: Annex-3 and Fig. 3). The average percentage increase of female medical students from 2004 to 2021 per year was 7.2% (CI 6.6–7.8%, *p* = 0.00) and 7.9% (CI 6.8–9.0%, *p* = 0.00) for male students.

When comparing citizenship, the average annual percentage increase of female Bahraini medical students from 2004 to 2021 was 8.4% (CI 6.8–10.0%, *p* = 0.00) and 6.4% (CI 6.2–6.7%, *p* = 0.00) for female non-Bahraini students. Trend analysis indicated that there were more female non-Bahraini students than female Bahraini students over the years; the difference being significantly higher in academic years 2020–21 compared to initial year (*p* = 0.03). Among Bahraini students, trend analysis showed that the number of females were comparatively higher than males, but there was no significant difference.

## Discussion

The discussion part is divided into three main sections; the leadership, workforce, and medical student to give a better insight on the women status and contribution in the kingdom of Bahrain and highlight the existing literature in the related fields.

### Leadership

Despite the increase in the number of females being enrolled into medical schools, women still struggle to shatter the glass ceiling to hold leadership positions [20–23]. Although improvements have been observed both locally and globally, permanent leadership positions are still held largely by men [24, 25]. For example, only 25% of African ministers of heath have been females and 24% of global health center directors at the top 50 US medical schools are females [26]. In Kuwait, the number of female heads of departments in several governmental hospitals has increased to reach a maximum of 73%, whereas the lowest rate reported in these hospitals was 44% [27].

In our study, leadership positions have been divided into multiple levels. Top administrative position which include Under Secretaries, Chief Executive Officers, Chiefs of Medical Staff, Heads of training Departments, Head of Departments, Head of Committees, and Academic Positions. Females make a 40.6% out of all these positions, whereas males make up 59.4%. There is a higher number of males in the heads of departments and heads of committees’ categories when compared to females (70.3% vs. 29.7% and 63.6% vs. 36.4%, respectively). Bahraini males and females equally dominate Academic position (Table 1). All leadership positions were dominated by Bahrain Females when compared to Non-Bahraini females.

### Physicians’ workforce

Among the diverse medical and surgical specialties, female physicians are still underrepresented in some specialties and more focused in others. It is well-documented in the literature that female physicians are underrepresented in several specialties: mainly the surgical specialties, such as general surgery, orthopedic surgery, neurosurgery, and emergency medicine [28–31]. Reasons for these low numbers of female physicians in certain specialties have been attributed to many factors including male dominance, physical demand, extended hours [29] family care [32], lack of support during pregnancy and in childcare [33], and lack of female role models [32]. On the other hand, female physicians are more focused in what is termed “people oriented” specialties, such as pediatrics, psychiatry [34, 35], and primary care [36].

At the time of the study, 3734 physicians were registered and licensed at NHRA out of whom 47.8% were males and 52.2% were females. Females considerably dominate the general license category compared to males (62.3% vs. 37.3%). However, the specialist and consultant license categories were dominated by male physicians rather than females (62.2% vs. 37.8% and 56.7% vs. 43.3%, respectively). Female Bahraini physicians at a specialist and consultant level slightly surpassed male Bahraini physicians (female to male: 11.9% vs. 10.4% and 33.2% vs. 30.4%, respectively). Further analysis revealed that Bahraini females held more consultant licenses than non-Bahraini female consultants (33.2% and 10.1%). On the other hand, non-Bahraini male and female physicians dominated the specialist license category compared to Bahraini physicians (51.8% vs. 10.4% and 26% vs. 11.9%).

Regarding specialties, our data showed that female physicians far exceed the number of males in primary care (75.7% vs. 24.3%) and Obstetrics and Gynecology (87.9% vs. 12.1%) only (Table 3). The higher number of female physicians in Obstetrics and Gynecology may be explained by cultural patient preferences. Being a conservative society, females still prefer to be examined by female physicians. There was also a larger number of females in family medicine. On the other hand, male consultants far exceeded females in all other specialties which is more significant in surgical specialties and orthopedic surgery. The surprising finding was the complete lack of female physician in intensive care (Table 3). However, this may be explained by two factors, one is that most intensive care units in Bahrain are run by anesthetists, and second, some physicians may have failed to renew their licenses on time and were, therefore, not included in the data as the data extracted only included physicians who hold valid licenses. Another specialty which completely lack female physicians is neurosurgery (Additional file 1: Table 5 Annex).

### Medical students

A review of the literature on gender distribution in medical schools shows a clear trend in which women overtly dominate graduate medical student seats, where this global phenomenon has already been seen in some European countries [37], the United States [38], Canada [39], and Australia [40]. In the Arab Gulf States, this is also seen in Kuwait [27] and Oman [41].

In this study, the number of medical students reflected similar trends with the number of female medical students exceeding the number of male medical students in Bahrain since 2004 (Additional file 1: Table 6 Annex). Most of the female medical students have been non-Bahraini. When considering only Bahraini medical students, female medical students far exceed their male counterparts.

The study reflects encouraging data regarding the status of women’s contribution to medicine in Bahrain. However, more focus needs to be directed toward female physicians to enable them to reach higher leadership positions. Mentorship and scholarships have been recommended in the literature to fill these gaps and achieve gender equity. Introducing the concept of mentorship in academic institutions with supportive interventions targeting gender equity can advance and retain women as they pursue their academic and professional careers, provided that these interventions are well-studied and take into account the variety of needs from women of diverse backgrounds, including nationality, socioeconomic status, cultural and educational background as well as other factors [42, 43].The concept of sponsorship programs has been applied in the business field and it is advocated that it can be applied to advance women in the academic medicine [44]. Sponsorship is defined as “the public support by a powerful, influential person for the advancement and promotion of an individual within whom he or she sees untapped or unappreciated leadership talent or potential” [44]. It is very common in the Arab Gulf States to provide governmental sponsorships for postgraduate studies abroad in various medical disciplines. No data has been published about governmental sponsorships of female physicians in Bahrain. One respectable example is King Abdullah Scholarship Program in Saudi Arabia which started in 2005 and sponsored distinguished academic Saudi residents, and the benefited number of women increased from 16% in 2005 to 44% in 2017 [45].

The study demonstrated that the graduation-to-work discrepancy in medicine for women in Bahrain is better than their male counterparts except in some leadership positions.

### Strengths and limitation

To our knowledge, this is the first study to assess women’s contribution in the medical field in the Kingdom of Bahrain. One limitation was that data was only collected from governmental institutions and the two medical colleges in the Kingdom.  As private medical facilities were not included in the study, women’s contribution in the private sector was not assessed. Second, the data from NHRA was collected at a specific time, reflecting the number of physicians with active valid licenses in one year only, which may not be an accurate estimate of actual numbers. Physicians who may have been in the process of renewing their license or those who had failed to renew their license were not included.

## Conclusions

The increase in the number of women in the medical community has been evident globally and the same trend is clearly reflected in the Kingdom of Bahrain. Although the number of males in leadership positions is slightly higher than that of females currently, with the continued growth in the number of female medical students and physicians, it is likely that these positions will be dominated by females in the future. What requires particular focus is the stark imbalance between male and female physicians in certain specialties. Females should be encouraged to enter training programs, where there is a severe shortage of females, and not be deterred from specializing in male dominated specialties. The results shown in this study could be used to direct decision makers to strive to balance the number of male and female physicians in some leadership posts and in residency training programs.

## Supplementary Information

**Additional file 1. ANNEX 1**. Medical specialties (Consultants & Specialists) breakdown by gender and citizenship.

**Additional file 2. ANNEX 2**. Surgical specialties (Consultants & Specialists) breakdown by gender and citizenship.

**Additional file 3. ANNEX 3**. Medical student graduates’ breakdown by gender and citizenship from 2004-05 to 2020-21.

## Data Availability

The data sets used and/or analysed during the current study are available from the corresponding author on reasonable request.

## Abbreviations

MOH: Ministry of Health
NHRA: National Health Regulatory Authority
SMC: Salmaniya Medical Complex
KHUH: King Hamad University Hospital
BDF–RMS: Bahrain Defence Force Royal Medical Services
AGU: Arabian Gulf University
RCSI–MUB: Royal College of Surgeons in Ireland–Bahrain
