# Supplementary material for: Women’s contribution to medicine in Bahrain: leadership and workforce
Source: Hum Resour Health. 2022 Sep 5;20:67. doi: 10.1186/s12960-022-00762-9 (PMC9444121; doi:10.1186/s12960-022-00762-9)
Supplement: Supplementary file 1 — Additional file 1. ANNEX 1. Medical specialties (Consultants & Specialists) breakdown by gender and citizenship. [file 12960_2022_762_MOESM1_ESM.docx]

**Women's Contribution to Medicine in Bahrain: Leadership and Workforce**

**ANNEX 1: Medical specialties (Consultants & Specialists) breakdown by gender and citizenship**

| **Medical Specialties** | **Female Number (%)** | | **Male Number (%)** | | **Total =**  **364** |
| --- | --- | --- | --- | --- | --- |
|  | **Bahraini** | **Non- Bahraini** | **Bahraini** | **Non-Bahraini** |  |
| Internal Medicine | 29 (26.9%) | | 79 (73.1%) | | 108 |
|  | 15 (13.8%) | 14 (13.1%) | 20 (18.5%) | 59 (54.6%) |  |
| Cardiology | 12 (22.7%) | | 41 (77.3%) | | 53 |
|  | 8 (15%) | 4 (7.7%) | 19 (35.8%) | 22 (41.5%) |  |
| Clinical genetics | 3 (100%) | | 0 (0%) | | 3 |
|  | 1 (33.3%) | 2 (66.7%) | 0 (0%) | 0 (0%) |  |
| Hematology/Hematology Oncology | 2 (33.4%) | | 4 (66.6%) | | 6 |
|  | 1 (16.7%) | 1 (16.7%) | 2 (33.3%) | 2 (33.3%) |  |
| Dermatology | 56 (60.9%) | | 36 (39.1%) | | 92 |
|  | 11 (11.9%) | 45 (49%) | 9 (9.7%) | 27 (29.4%) |  |
| Endocrinology | 4 (36.4%) | | 7 (63.6%) | | 11 |
|  | 4 (36.4%) | 0 (0%) | 5 (45.4%) | 2 (18.2%) |  |
| Forensic medicine | 0 (0%) | | 3 (100%) | | 3 |
|  | 0 (0%) | 0 (0%) | 0 (0%) | 3 (100%) |  |
| Aviation /Hyperbaric Medicine | 1 (20%) | | 4 (80%) | | 5 |
|  | 1 (20%) | 0 (0%) | 4 (80%) | 0 (%) |  |
| Gastroenterology | 3 (14.3%) | | 18 (85.7%) | | 21 |
|  | 3 (14.3%) | 0 (0%) | 11 (52.3%) | 7 (33.4%) |  |
| Infectious disease | 1 (33.4%) | | 2 (66.6%) | | 3 |
|  | 0 (0%) | 1 (33.4%) | 2 (66.6%) | 0 (0%) |  |
| Nephrology | 2 (11.8%) | | 15 (88.2%) | | 17 |
|  | 2 (11.8%) | 0 (0%) | 6 (35.2%) | 9 (53%) |  |
| Neurology | 3 (15.8%) | | 16 (84.2%) | | 19 |
|  | 2 (10.5%) | 1 (5.3%) | 6 (31.5%) | 10 (52.7%) |  |
| Pulmonary | 4 (28.6%) | | 10 (71.4%) | | 14 |
|  | 1 (7.1%) | 3 (21.5%) | 4 (28.5%) | 6 (42.9%) |  |
| Rheumatology | 4 (50%) | | 4 (50%) | | 8 |
|  | 2 (25%) | 2 (25%) | 2 (25%) | 2 (25%) |  |
| Tropical medicine | 0 (0%) | | 1 (100%) | | 1 |
|  | 0 (0%) | 0 (0%) | 0 (0%) | 1 (100%) |  |
