# Supplementary material for: Women’s contribution to medicine in Bahrain: leadership and workforce
Source: Hum Resour Health. 2022 Sep 5;20:67. doi: 10.1186/s12960-022-00762-9 (PMC9444121; doi:10.1186/s12960-022-00762-9)
Supplement: Supplementary file 2 — Additional file 2. ANNEX 2. Surgical specialties (Consultants & Specialists) breakdown by gender and citizenship. [file 12960_2022_762_MOESM2_ESM.docx]

**Women's Contribution to Medicine in Bahrain: Leadership and Workforce**

**ANNEX 2: Surgical specialties (Consultants & Specialists) breakdown by gender and citizenship**

| **Surgical Specialties** | **Female Number (%)** | | **Male Number (%)** | | **Total =**  **352** |
| --- | --- | --- | --- | --- | --- |
|  | **Bahraini** | **Non- Bahraini** | **Bahraini** | **Non-Bahraini** |  |
| Ophthalmology | 28 (36.4%) | | 49 (63.6%) | | 77 |
|  | 19 (24.6%) | 9 (11.8%) | 24 (31.1%) | 25 (32.5%) |  |
| ENT | 16 (23.6%) | | 52 (76.4%) | | 68 |
|  | 13 (19.1%) | 3 (4.5%) | 25 (36.7%) | 27 (39.7%) |  |
| General surgery | 13 (12.7%) | | 90 (87.3%) | | 103 |
|  | 8 (7.7%) | 5 (5%) | 31 (30%) | 59(57.3%) |  |
| Neurosurgery | 0 (0%) | | 15 (100%) | | 15 |
|  | 0 (0%) | 0 (0%) | 4 (26.6%) | 11 (73.4%) |  |
| Urology | 2 (6.1%) | | 31 (93.9%) | | 33 |
|  | 2 (6.1%) | 0 (0%) | 17 (51.5%) | 14 (42.4%) |  |
| Vascular/ Cardiothoracic Surgery | 1 (5.9%) | | 16 (94.1%) | | 17 |
|  | 0 (0%) | 1 (5.9%) | 9 (52.9%) | 7 (41.2%) |  |
| Plastic surgery | 6 (20%) | | 24 (80%) | | 30 |
|  | 3 (10%) | 3 (10%) | 7 (23.3%) | 17 (56.7%) |  |
| Pediatric surgery | 1 (11.2%) | | 8 (88.8%) | | 9 |
|  | 1 (11.2%) | 0 (0%) | 5 (55.5%) | 3 (33.3%) |  |
