# Supplementary material for: Women’s contribution to medicine in Bahrain: leadership and workforce
Source: Hum Resour Health. 2022 Sep 5;20:67. doi: 10.1186/s12960-022-00762-9 (PMC9444121; doi:10.1186/s12960-022-00762-9)
Supplement: Supplementary file 3 — Additional file 3. ANNEX 3. Medical student graduates’ breakdown by gender and citizenship from 2004-05 to 2020-21. [file 12960_2022_762_MOESM3_ESM.docx]

**Women's Contribution to Medicine in Bahrain: Leadership and Workforce**

**ANNEX 3: Medical student graduates’ breakdown by gender and citizenship from 2004-05 to 2020-21**

|  | **Female number (%)** | | **Male number (%)** | | **Total** |
| --- | --- | --- | --- | --- | --- |
|  | **Female Bahraini** | **Female non-Bahraini** | **Male Bahraini** | **Male non-Bahraini** |  |
| **2004-05** | 438 (66.1%) | | 225 (33.9%) | | 663 |
|  | 130 (19.6%) | 308 (46.5%) | 52 (7.8%) | 173 (26.1%) |  |
| **2005-06** | 506 (64.1%) | | 283 (35.9%) | | 789 |
|  | 166 (21.0%) | 340 (43.1%) | 63 (8.0%) | 220 (27.9%) |  |
| **2006-07** | 576 (63.5%) | | 331 (36.5%) | | 907 |
|  | 211 (23.3%) | 365 (40.2%) | 88 (9.7%) | 243 (26.8%) |  |
| **2007-08** | 642 (63.3%) | | 373 (36.7%) | | 1015 |
|  | 243 (23.9%) | 399 (39.3%) | 107 (10.5%) | 266 (26.2%) |  |
| **2008-09** | 689 (61.6%) | | 430 (38.4%) | | 1119 |
|  | 276 (24.7%) | 413 (36.9%) | 143 (12.8%) | 287 (25.6%) |  |
| **2009-10** | 735 (59.4%) | | 503 (40.6%) | | 1238 |
|  | 284 (22.9%) | 451 (36.4%) | 155 (12.5%) | 348 (28.1%) |  |
| **2010-11** | 760 (59.6%) | | 515 (40.4%) | | 1275 |
|  | 286 (22.4%) | 474 (37.2%) | 152 (11.9%) | 363 (28.5%) |  |
| **2011-12** | 806 (59.6%) | | 546 (40.4%) | | 1352 |
|  | 317 (23.4%) | 489 (36.2%) | 151 (11.2%) | 395 (29.2%) |  |
| **2012-13** | 854 (58.1%) | | 616 (41.9%) | | 1470 |
|  | 344 (23.4%) | 510 (34.7%) | 183 (12.4%) | 433 (29.5%) |  |
| **2013-14** | 935 (57.7%) | | 686 (42.3%) | | 1621 |
|  | 379 (23.4%) | 556 (34.3%) | 219 (13.5%) | 467 (28.8%) |  |
| **2014-15** | 1018 (58.5%) | | 721 (41.5%) | | 1739 |
|  | 418 (24.0%) | 600 (34.5%) | 233 (13.4%) | 488 (28.1%) |  |
| **2015-16** | 1109 (59.1%) | | 766 (40.9%) | | 1875 |
|  | 451 (24.1%) | 658 (35.1%) | 255 (13.6%) | 511(27.3%) |  |
| **2016-17** | 1163 (60.4%) | | 764 (39.6%) | | 1927 |
|  | 474 (24.6%) | 689 (35.8%) | 277 (14.4%) | 487 (25.3%) |  |
| **2017-18** | 1204 (60.6%) | | 782 (39.4%) | | 1986 |
|  | 503 (25.3%) | 701 (35.3%) | 303 (15.3%) | 479 (24.1%) |  |
| **2018-19** | 1265 (62.0%) | | 775 (38.0%) | | 2040 |
|  | 496 (24.3%) | 769 (37.7%) | 285 (14.0%) | 490 (24.0%) |  |
| **2019-20** | 1285 (62.0%) | | 788 (38.0%) | | 2073 |
|  | 479 (23.1%) | 806 (38.9%) | 269 (13.0%) | 519 (25.0%) |  |
| **2020-21** | 1335 (62.8%) | | 791 (37.2%) | | 2126 |
|  | 472 (22.2%) | 863 (40.6%) | 240 (11.3%) | 551 (25.9%) |  |
